# Estimating the prevalence of problem drug use from drug-related mortality data

**DOI:** 10.1111/add.15111

**Published:** 2020-06-09

**Authors:** Hayley E. Jones, Ross J. Harris, Beatrice C. Downing, Matthias Pierce, Tim Millar, A. E. Ades, Nicky J. Welton, Anne M. Presanis, Daniela De Angelis, Matthew Hickman

**Affiliations:** 1 Population Health Sciences, Bristol Medical School, University of Bristol, Bristol, UK; 2 Centre for Infectious Disease Surveillance and Control, Public Health England, London, UK; 3 Division of Psychology and Mental Health, School of Health Sciences, University of Manchester, Manchester, UK; 4 MRC Biostatistics Unit, University of Cambridge, Cambridge, UK

**Keywords:** Bayesian analysis, capture–recapture, hidden populations, indirect estimation, multiplier methods, synthetic estimation

## Abstract

**Background and Aims:**

Indirect estimation methods are required for estimating the size of populations where only a proportion of individuals are observed directly, such as problem drug users (PDUs). Capture–recapture and multiplier methods are widely used, but have been criticized as subject to bias. We propose a new approach to estimating prevalence of PDU from numbers of fatal drug-related poisonings (fDRPs) using linked databases, addressing the key limitations of simplistic ‘mortality multipliers’.

**Methods:**

Our approach requires linkage of data on a large cohort of known PDUs to mortality registers and summary information concerning additional fDRPs observed outside this cohort. We model fDRP rates among the cohort and assume that rates in unobserved PDUs are equal to rates in the cohort during periods out of treatment. Prevalence is estimated in a Bayesian statistical framework, in which we simultaneously fit regression models to fDRP rates and prevalence, allowing both to vary by demographic factors and the former also by treatment status.

**Results:**

We report a case study analysis, estimating the prevalence of opioid dependence in England in 2008/09, by gender, age group and geographical region. Overall prevalence was estimated as 0.82% (95% credible interval = 0.74–0.94%) of 15–64-year-olds, which is similar to a published estimate based on capture–recapture analysis.

**Conclusions:**

Our modelling approach estimates prevalence from drug-related mortality data, while addressing the main limitations of simplistic multipliers. This offers an alternative approach for the common situation where available data sources do not meet the strong assumptions required for valid capture–recapture estimation. In a case study analysis, prevalence estimates based on our approach were surprisingly similar to existing capture-recapture estimates but, we argue, are based on a much more objective and justifiable modelling approach.

## Introduction

Information on the size of the injecting and opioid-using population globally is increasingly important to support and motivate public health strategies to eliminate hepatitis C virus (HCV) [1–5], reverse upward trends in opioid overdose deaths [6,7] and monitor treatment provision [7,8]. It is well known that population surveys seriously underestimate the prevalence of problem drug use—although they can contribute information in other ways [9,10]. Instead, ‘indirect’ methods are recommended, such as capture–recapture and multipliers. *Addiction* has previously highlighted the growing dissatisfaction with the quality and transparency in the evidence base on the prevalence of opioid use and injecting [11].

There are discrepancies in global estimates of the prevalence of injecting and other problem drug use, as well as inconsistencies in and controversies concerning national and city-level estimates. For instance, it is unclear what is the original method or source data for US estimates [8,12–14]. Recent estimates in Australia were acknowledged to be potentially biased and were challenged by other researchers [15–18]. National estimates of injecting drug use were found to be inconsistent with information on HCV morbidity in England, and discrepant estimates have been generated in both Scotland and England based on the same source data [1,19–22].

Indirect methods of prevalence estimation rely upon assumptions that are often violated, and can be impossible to validate in any given data set. Broadly speaking, the validity of capture–recapture analysis is questionable when the dependence structure between the data sources used is complex, and when the target population is heterogeneous in behaviour [23,24]. In this situation, it can be impossible to distinguish between a range of models with similar or identical fit which may, however, give widely different prevalence estimates. See Box 1 for more detail and a case study.

Multiplier methods are a simple and widely used alternative [25–31]. Estimation of the size of the target population (N) requires a ‘benchmark’ (d)—the number of individuals from the target population that are detected via some source—and an estimate of the proportion of the target population that we would expect to detect in this way (p); N is then estimated by *d/p*, where *1/p* is often referred to as the ‘multiplier’. For example, dividing the number of fatal drug-related poisonings (fDRPs) among problem drug users (PDUs) over a year by an estimate of the proportion of PDUs who die such a death in 1 year gives an estimate of the number of PDUs. The approach has its basis in sampling theory and can be described more generally as inverse probability weighting, or a simplified special case of the back-calculation approach [32]. It is strongly related to the case of a two-sample capture–recapture analysis and generally viewed as inferior to multiple source (three or more sources) capture–recapture. Where a ‘perfect’ multiplier is not available, pragmatism may favour a multiplier that is neither specific to the benchmark population nor year to which it is being applied [13,15,33–34], which can dramatically affect the population estimate [35–37]. It is difficult to then judge the validity of the resulting population size estimates.

In this paper we revisit the idea of estimating prevalence from drug-related mortality data. We propose a new methodological approach within a Bayesian statistical framework, addressing the most common criticisms of traditional ‘multiplier’ approaches.

## Limitations of Traditional Multiplier Methods

We first describe several common criticisms of multiplier methods, which we aim to address with our proposed approach. We focus on mortality multipliers in this text, but the same general criticisms apply to other types of multipliers, e.g. treatment multipliers.

(i) *Bias due to borrowing multipliers across settings:* to be unbiased, the multiplier needs to be estimated from a representative and contemporaneous sample of the target population. Rates of events such as fDRP can be expected to vary across locations and over time, such that ‘borrowing’ multipliers across settings will not often be valid. Further, different studies may use different working definitions of fDRP.
(ii) *Failure to account for heterogeneity in the multiplier:* even within the time-period and location of interest, fDRP rates are known to vary by age group and gender [45–47], with the two factors probably interacting [22] and, importantly, to vary by drug treatment status [24,48,49]. Traditional multiplier exercises have ignored such heterogeneity.
(iii) *Inappropriate case definitions:* multiplier approaches assume that all individuals in the ‘benchmark’ were among the target population. This can be difficult to establish in practice. For example, not all fDRP deaths occur among PDUs. Further, it is crucial that the same case definition (e.g. of fDRP) is used to derive both the benchmark and the multiplier.
(iv) *Failure to adequately account for uncertainty:* there are two sources of statistical uncertainty: sample uncertainty in the benchmark, *d*, and uncertainty around the multiplier. Both should be accounted for in the calculation of an appropriate confidence or credible interval (CrI) around the prevalence estimate. Where intervals do not account for both sources of uncertainty, estimates are susceptible to over-interpretation.
(v) *Imprecise estimates:* if the event is relatively rare (e.g. fDRP) and uncertainty is appropriately accounted for, the CrI may be extremely wide, and potentially uninformative for planning and decision-making. For example, we identified *d* = 15 fDRPs among people who inject drugs in Bristol, England in 2011. We accounted for uncertainty both in *d* and our estimate of the relevant fDRP rate in a Bayesian statistical framework, but subsequently the CrI around the prevalence estimate was very wide: 0.9% (95% CrI = 0.4–1.6%) [23].

## Case Study Data

We provide a case study of estimation of the number of opioid-dependent people aged 15–64 in England in the financial year 2008/09. We focus on 2008/09 because a large fully linked data set was available through the Drug Data Warehouse (DDW), and this was the last year of linkage. The DDW contains anonymized linked data on all opioid-dependent people in contact with treatment services or identified through criminal justice services in England between April 2005 and March 2009 [46,50]. Critically for this estimation exercise, all identified cases were linked to official mortality records. The treatment records include dates on which each individual started and ceased each type of treatment, from which we categorized all periods as ‘on’ or ‘off’ opioid substitution treatment. This allowed us to estimate fDRP rates during periods on versus off treatment [24]. One difficulty, however, is that linkage was based only on initials and dates of birth. It is therefore expected that some fDRPs occurring within the DDW sample were missed. For further details of the linkage process and its limitations, see Pierce [51].

We produce stratified estimates of prevalence by gender, age group (15–34 and 35–64 years) and by nine geographical regions. We index group (gender/age/region combination) by *g* = 1,…,36. We denote the number of opioid-dependent people observed in the DDW in 2008/09 in each group by $n_{g}^{DDW}$ and total (unknown) prevalence in each group by *π_g_*. By definition, $\pi_{g}=\left(n_{g}^{DDW}+n_{g}^{miss}\right)/P_{g}$, where *P_g_* is the total population size of group *g* and $n_{g}^{miss\;}$ is the number of opioid-dependent people in group *g* who were not observed in the DDW cohort.

For the purposes of this case-study analysis, we use a restricted definition of fDRP. We model only deaths with underlying cause coded with ICD10 code F11 (‘Mental and behavioural disorders due to use of opioids’) or F19 (‘Mental and behaviour disorders due to multiple drug use and use of other psychoactive substances’), in which at least one opioid was explicitly mentioned on the death certificate. F11 and F19 are the categories most likely to relate to long-term dependent drug use. Herein, we will use the term fDRP to refer to these deaths specifically. Note that this is a narrower definition than used in our simpler analysis in Bristol [24] described above (see ‘Imprecise estimates’ section); therefore, the estimated rates of fDRPs are not comparable across these analyses.

In 2008/09, there were 181 fDRPs linked to the 176 329 opioid-dependent people in the DDW (Table 1). Following stratification by the 36 groups and also by treatment status (‘on’ versus ‘off’), there were zero fDRPs in several cells. This presents difficulties for estimation of fDRP rates by group and treatment status. To address this issue, we obtained data of the same form for each of the 4 years of the DDW, allowing us to estimate fDRP rates more precisely. Summary data for all 4 years are shown in Table 1.

In addition to the DDW data, we obtained a data extract from the Office for National Statistics (ONS) specifying all fDRPs in 2008/09. For each group *g*, we subtracted the number of fDRPs linked to DDW individuals, $d_{g}^{DDW}$, from the total number according to the ONS data extract, $d_{g}^{all\;}$, to obtain the number not linked to the DDW cohort: $d_{g}^{miss\;}=d_{g}^{all\;}-d_{g}^{DDW}$. In total, 273 fDRPs in 2008/09 were not linked to the DDW sample (Table 1).

For comparison with what follows, we first provide a crude estimate of the population size based on these data, using a Bayesian version of a simple ‘mortality multiplier’ [23]. Aggregating across treatment status and group, the observed fDRP rate in 2008/09 was 1.22 per 1000 person-years. Using the total number of fDRP in 2008/09 (454) as a single benchmark, we arrive at an estimate of 372 400 (95% CrI = 313 900–444 100) opioid-dependent people, or a prevalence of 1.08% (0.91–1.29%). This simple approach does not, however, allow for bias in the estimate of the fDRP rate due to imperfect matching, or for heterogeneity in fDRP rate.

## Bayesian Mortality-Based Prevalence Estimation

In this section we describe our approach to modelling the DDW and ONS data together, simultaneously estimating models for fDRP rates and for prevalence of opioid dependence.

### General modelling approach

The essence of our approach is as follows. We use the DDW data to estimate fDRP rates among opioid-dependent people during periods ‘on’ and ‘off’ treatment in 2008/09, by group. We assume that fDRP rates during periods out of treatment derived from this sample also apply to the unobserved opioid-dependent people who are, by definition, not in treatment. These mortality rates, together with a regression model for prevalence, determine our estimates of each $n_{g}^{miss}$ or, equivalently, of prevalence. Our model also incorporates a correction for incomplete matching of DDW records to mortality records.

We take a Bayesian approach to estimation using Markov chain Monte Carlo (MCMC) simulation, because this enables the computational flexibility required to estimate all parameters simultaneously. The key difference between a Bayesian and frequentist approach is the specification of prior beliefs on parameters being estimated. ‘Uninformative’ prior distributions lead to parameter estimates that are driven by the observed data, and would correspond to results obtained from a frequentist approach if it were possible to compute it. We use uninformative prior distributions wherever possible (for more details see the ‘Model for fDRP rate among opioid-dependent people’ section).

We present an overview of the model in sections ‘Model for fDRP rate among opioid-dependent people’ and ‘Model for fDRPs not linked to the DDW data’. Full details are provided in the Supporting information, Appendix B. WinBUGS [52] code used to fit the model is presented in Supporting information, Appendix C.

### Model for fDRP rate among opioid-dependent people

We fitted mixed-effect Poisson regression models to observed fDRPs in the DDW, with person-years at risk as offsets. Although the prevalence estimation requires only estimates of rates during periods out of treatment in 2008/09, we modelled all 4 years of mortality data and deaths during periods on as well as off treatment. Our motivation was to borrow strength through some sharing of parameters, given the sparse mortality data.

The regression model included the following explanatory variables: region, gender, age group, treatment status and year. As year was included as a covariate, the model allowed for changes in fDRP rates over time. Region was modelled as a random effect due to small counts. We used the deviance information criterion (DIC) to explore support for inclusion of interactions between these explanatory variables. Lower values of the DIC indicate a better fit after penalizing for model complexity [53]. The DIC supported inclusion of gender × age group, treatment × age and year × treatment interaction terms, which were therefore included in the primary analysis. Although there is substantive rationale for the first two of these, we questioned the plausibility of ‘treatment effects’ on mortality changing considerably over a 4-year period. In a sensitivity analysis, we therefore explored the impact of excluding the year × treatment interaction term.

An additional offset *‘pmatch’* was included in the regression model, representing the probability that any fDRP among the DDW sample was correctly identified through linkage. This parameter was the only variable assigned an informative prior distribution in our Bayesian analysis: this was necessary, as it would be impossible to estimate this from the data. We assumed that between 74 and 84% of DDW deaths were correctly matched to ONS records. This was based on examining DDW data on a cohort of individuals identified in treatment records as ‘discharged as dead’ and checking the proportion of these that were correctly identified by the linkage process. Inclusion of *‘pmatch’* in the model means that estimated fDRP rates are corrected for imperfect matching to ONS records. Otherwise, rates would be underestimated, subsequently leading to over-estimates of prevalence. To demonstrate the impact of the adjustment for imperfect linkage, we also provide results from an analysis with *‘pmatch’* set to 1.

**Figure 1:**
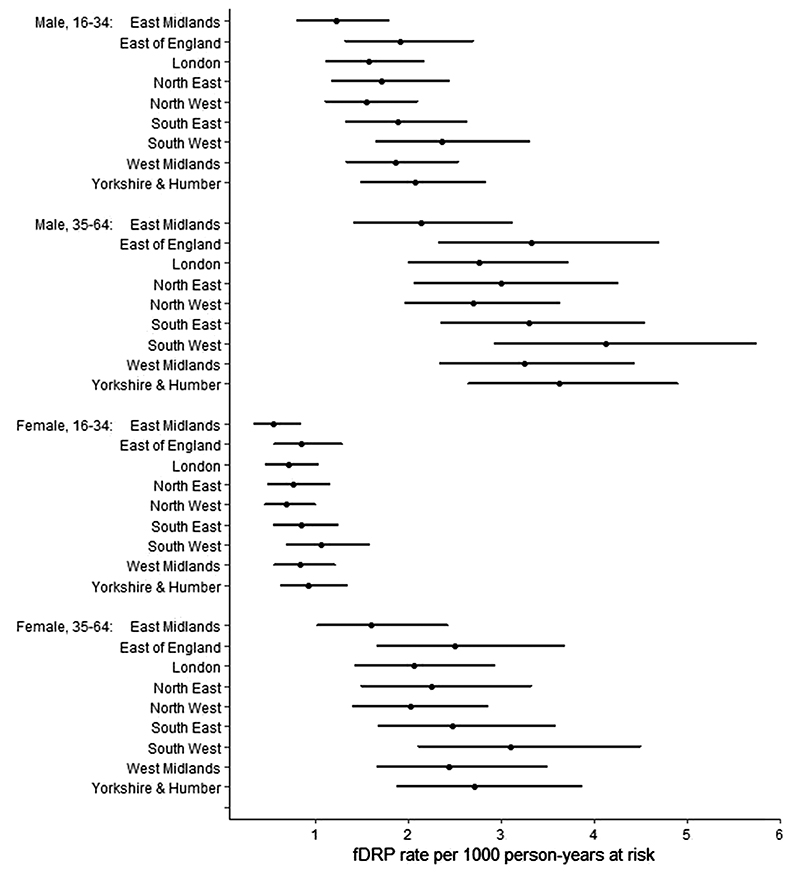
Estimated fatal drug-related poisoning (fDRP) rates* during periods out of treatment, with 95% credible intervals. *Using our restricted definition for modelling purposes: see Case study data section

Estimated ‘off treatment’ fDRP rates in each group are displayed in Fig. 1.

### Model for fDRPs not linked to the DDW data

We assume Poisson distributions for the number of fDRPs not linked to the DDW in each group, $d_{g}^{miss}$. We assume that the unobserved opioid-dependent people $\left(n_{g}^{miss\;}\right)$ had a mortality rate equal to the ‘off treatment’ mortality rate for the relevant group, estimated from the model above. We added an additional term to the expected mean of each $d_{g}^{miss}$ to account for the proportion, *1-pmatch*, of fDRPs occurring within the DDW sample that were missed by the linkage process.

### Model for prevalence of opioid dependence

Finally, we specified a regression model for the prevalence of opioid dependence in each group. We assumed a linear model for logit-transformed prevalence, with age, gender and region effects. We also included age × gender, gender × region and age × region interaction terms. Region-specific intercept terms were unconstrained, while interactions involving region were modelled as random effects.

**Figure 2:**
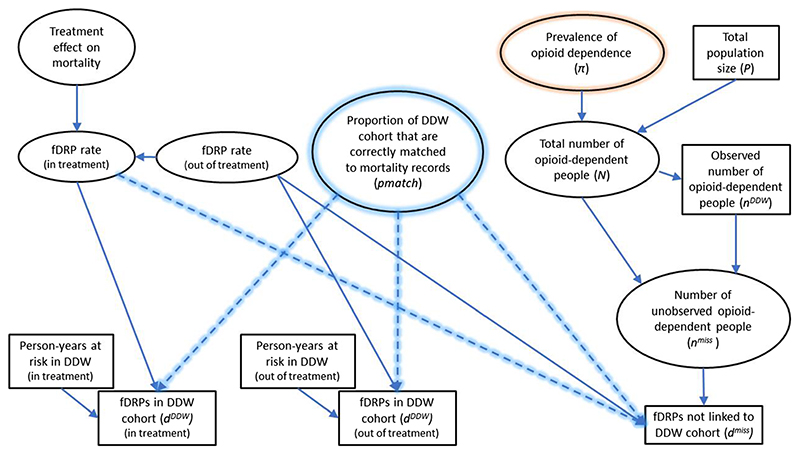
Schematic diagram showing the relationships between data sources (rectangles) and parameters (ellipses). This is a simplification of the full model, which also incorporates gender, age group and region effects. Dashed lines with blue shading indicate the correction for imperfect matching of Drug Data Warehouse (DDW) records to the Office of National Statistics (ONS) mortality register. The parameter *‘pmatch’* is given an informative prior distribution (see text). Orange shading highlights the key parameter(s) of interest to be estimated, i.e. prevalence of opioid dependence (π). fDRP =fatal drug-related poisoning. [Colour figure can be viewed at wileyonlinelibrary.com]

A schematic diagram showing the relationships between the data sources and parameters is provided in Fig. 2.

### Addressing the limitations of the multiplier method

Our approach addresses each of the difficulties described with traditional multiplier approaches in the ‘Limitations of traditional multiplier methods’ section.

(i) *Bias due to borrowing multipliers across settings:* we minimize potential bias in estimates of fDRP rates by estimating these from contemporaneous data in England. The DDW sample is very large, and can be expected to include a wide range of opioid-dependent people and observation periods on and off treatment. We also allow for an anticipated slight downward bias in estimated fDRP rates from DDW data, due to imperfect linkage to mortality records (see the ‘Model for fDRP rate among opioid-dependent people’ section).
(ii) *Failure to account for heterogeneity in the multiplier:* a key feature of our analysis is that we recognize that opioid-dependent people not observed in the DDW were, by definition, not in treatment, as the DDW includes all contact with treatment services. As such, the mortality rates that we apply to the unobserved fDRPs to estimate prevalence [after allowing for some misclassification, as described in (i)] are estimated rates during periods out of treatment: substantially higher than rates during treatment [24]. Failure to account for this would lead to a potentially large over-estimation of prevalence. Further, we allow for heterogeneity in fDRP rates by gender, age group and region.
(iii) *Inappropriate case definitions:* our restricted definition of fDRP limits the possibility of the ‘benchmark’ number of deaths including individuals outside the target population. We use a consistent definition of fDRP across both parts of the model.
(iv) *Failure to adequately account for uncertainty:* we use an MCMC simulations-based estimation approach, in which each source of sampling uncertainty is automatically propagated to the final prevalence estimates, providing appropriate 95% CrIs.
(v) *Imprecise estimates:* direct linkage of a large sample of opioid-dependent people to mortality records enabled us to specifically estimate the undercount of the DDW, which we then (essentially) added to the total observed. This approach substantially reduces uncertainty in our final estimates, and is justifiable given that it is known with certainty that there are at least as many opioid-dependent people as observed.

## Case Study Results

In total, we estimated there were 106 700 (95% CrI = 78 200–147 900) ‘unobserved’ opioid-dependent people in England in 2008/09. This corresponds to an estimated total number of opioid-dependent people of 283 100 (95% CrI = 254 600–324 200), or a prevalence of 0.82% (0.74–0.94%) among 15–64-year-olds in England. We note that the total number of opioid-dependent people in treatment during 2008/09 was 151 600 (data from the DDW: not shown); therefore, our results imply that the total proportion in treatment at some point during the year was 46% (41–51%).

**Figure 3:**
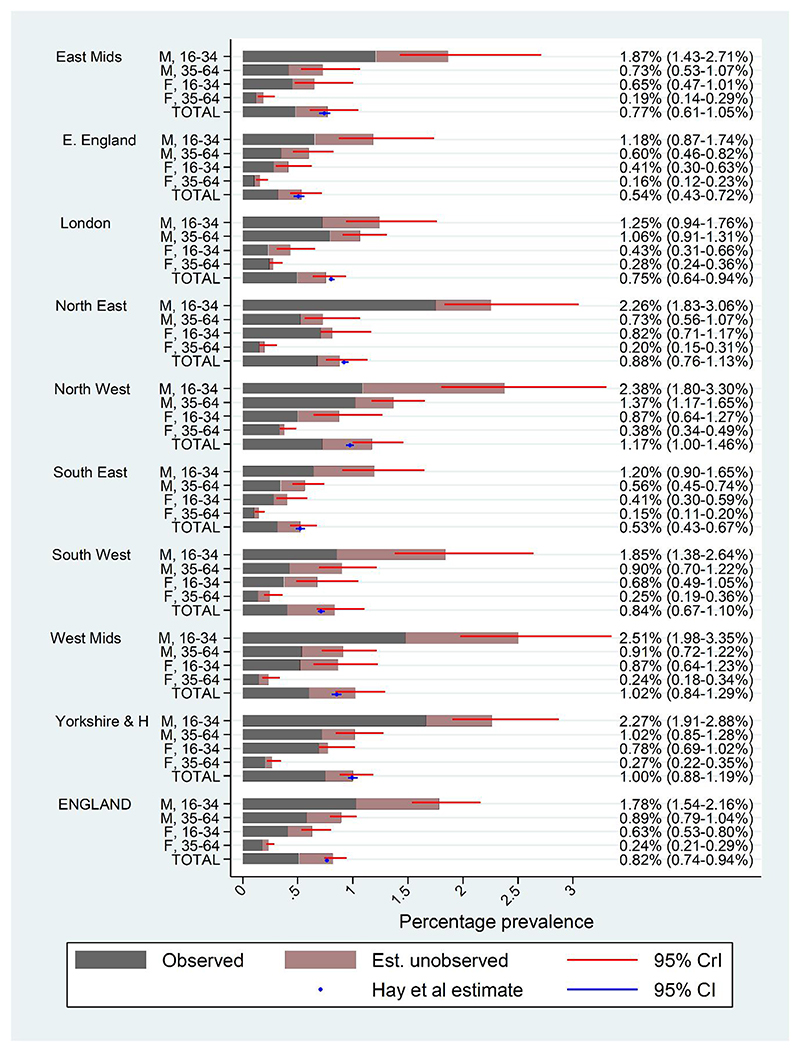
Estimated prevalence (with 95% credible intervals, CrIs) of opioid dependence in England in 2008/09, by gender, age group and geographical region. For comparison, estimates from Hay *et al*. [40] with 95% confidence intervals (CI) are shown in blue. [Colour figure can be viewed at wileyonlinelibrary.com]

Prevalence estimates by gender, age group and region are shown in Fig. 3. Despite the sparse mortality data by region, we see that regional differences in prevalence can be estimated. Across regions, overall prevalence estimates ranged from 0.53% (0.43–0.67%) in the South East to 1.17% (1.00–1.46%) in the North West. Group-specific estimates ranged from 0.15% (0.11–0.20%) in older females in South East England to 2.51% (1.98–3.35%) in younger men in the West Midlands.

Estimated patterns by age group and gender were as expected, with prevalence being estimated to be lower in older and female populations. There was evidence for moderate variation in the age effect across regions [standard deviation (SD) of random effects = 0.36, 95% CrI = 0.18–0.78]: the difference in prevalence by age group is estimated to be smallest in London and greatest in the North East. Regional variation in gender effects on prevalence was smaller (SD = 0.07, 95% CrI = 0.00–0.28).

A sensitivity analysis in which we excluded the year × treatment interaction term from the mortality model provided higher estimates of fDRP rates in the untreated group in 2008/09, which resulted in a somewhat lower overall prevalence estimate of 0.78% (0.72–0.87%) or *n* = 267 300 (245 900–298 100).

The impact of the adjustment for matching is considerable: setting the parameter *‘pmatch’* to 1 (in the primary model) produced a higher overall prevalence estimate of 0.96% (0.86–1.09%) or *n* = 328 800 (296 200–373 900).

For comparison with our estimates, in Fig. 3 we also display the official estimates of the prevalence of opioid dependence in England in 2008/09, based on capture–recapture estimation [40] (see Discussion).

## Discussion

### Main findings

We have described an alternative prevalence estimation method that utilizes linkage between drug treatment or other records and mortality registers, addresses the main limitations of traditional ‘multipliers’ and can be used when suitable information and data sources for capture–recapture analysis are unavailable.

Our Bayesian modelling approach involves simultaneously fitting two regressions—for mortality rates and prevalence—allowing for systematic differences in both factors by gender, age group and region, as well as dependence of fDRP rates on opioid substitution treatment status. The method also allows us to incorporate information on misclassification, so that undercounts of deaths in the observed population can be accounted for. The impact of these factors is demonstrated in our case study where, in a crude analysis ignoring both of these factors, prevalence was estimated as 1.08%, compared with 0.96% in an analysis accounting for heterogeneity in fDRP rate and prevalence but not for imperfect matching, and 0.82% in our (primary) analysis accounting for both factors.

### Limitations

We recognize, however, a number of potential limitations with our approach. First, the method relies upon a rich data set that can provide contemporaneous estimates of mortality rates, so that we avoid the rarely justifiable assumption that a ‘multiplier’ from another location or time is applicable to our target population. In our case study, we made use of the DDW cohort, which linked all people reported to be in opioid substitution treatment, and other administrative data sources that assessed opioid dependence, with the national mortality register [24,46,54]. Clearly, establishing the mechanisms and permissions for such linkage requires investment. However, this investment is essential for understanding and monitoring drug-related mortality. Historically, linked drug treatment and mortality data were rare. However, linked health data are becoming much more common and central to epidemiology among multiple fields, including addiction. For example, there were 124 mortality cohort studies in a recent systematic review, most of which began during the last 10 years [55]. Our method offers further motivation for collecting data on drug-related mortality, which we also note is a comparatively small step if data on people entering drug treatment are already being collected [48,49,56].

Secondly, to minimize the possibility of the deaths modelled being outside the target population (i.e. deaths in non-opioid-dependent people), we narrowed the definition of ‘fDRP’ for modelling purposes to F11 and F19 codes with opioids specified on the death certificate. The drawback of this was that the number of deaths to be modelled was relatively small, reducing statistical power. As a result, in this case study we were unable to estimate prevalence at lower geographical levels. This might be feasible if a broader working definition of fDRP could be used: this would require more information on the probable proportion of deaths by ICD10 code occurring in the target population. Notably the inclusion of random effects in the model allows for ‘borrowing of strength’ across subgroups of the population.

Thirdly, our estimates are dependent upon one main assumption: that, within each group *g*, fDRP rates among unobserved people with opioid disorders are equal to rates among those observed in the DDW sample during periods out of treatment. We believe this is justifiable for older opioid-dependent people in the United Kingdom (and many other developed countries), as most people are likely to enter drug treatment at some point [57]. Further, the DDW sample includes some individuals who were not in treatment during the 4-year sampling period [46]. Nonetheless, we note that our model averages the excess risk of overdose out of treatment and does not specifically account for elevated mortality risk during the first 4 weeks following treatment cessation [24], nor for potential differences in fDRP rates by duration of treatment or pattern of treatment history [58,59]. The modelling approach could be further developed to allow for such factors. The key underlying assumption could also be tested if more data sources were incorporated within the Bayesian model in a multi-parameter evidence synthesis framework [60,61].

Finally, our case study results are dependent upon an informative prior distribution that we derived for ‘*pmatch*’, the probability that a fDRP among the DDW sample was correctly identified by linkage. However, this seems preferable to assuming that linkage is perfect (corresponding to setting an extremely strong prior of *pmatch* = 1 with certainty). Ideally, we would have better-quality empirical data on matching to incorporate into the modelling. We note that imperfect linkage is also a common difficulty in many capture–recapture exercises. For example, the annual estimation exercise in England is similarly based on matching by only initials, date of birth and gender, and this is not accounted for in analysis [39].

### Other evidence

Capture–recapture is a widely used alternative approach to prevalence estimation. However, when the dependence structure across data sources is very complex, and the target population heterogeneous, we have found capture–recapture estimates to not be robust: multiple models with similar fit can produce widely different prevalence estimates (Supporting information, Appendix A, [22]). At a local level, careful consideration of the nature of referrals between services and incorporation of additional information may inform choice between competing models [24]. If, however, there is insufficient information to choose between models, alternative estimation approaches are needed. Bayesian model-averaging techniques have been proposed to avoid having to choose between models [21]. However, we do not believe that averaging across competing models producing widely different estimates offers a scientifically justifiable solution.

In our case study analysis we found that our national and regional estimates of the prevalence of opioid dependence were similar to published estimates derived through capture–recapture, but with wider uncertainty intervals [40]. However, these capture–recapture estimates were based on aggregating results from more than 150 stratified analyses, unjustified assumptions and repeated model-fitting using different approaches until the estimates were deemed credible by the investigators (see Appendix A). Model selection based on credibility of the results alone is not a valid or replicable approach [23,24].

### Implications

Understanding and monitoring drug-related mortality requires linkage of drug treatment and other administrative data sets to mortality registers. We demonstrate a method that can utilize these linked data to estimate the prevalence of opioid disorders.

Our method is an initial step to developing a coherent approach to estimate prevalence of opioid disorders. The model could be extended to estimate injecting opioid use and to model changes over time, in addition to incorporating additional data sources in a multi-parameter evidence synthesis framework, allowing the consistency of evidence to be more formally assessed. Estimates of drug-related harms such as HIV and HCV are reliant upon the assumption that estimates of the prevalence of people who inject drugs are unbiased [1,62,63]. Our model provides an alternative and potentially more justifiable approach than the current annual capture–recapture exercises in the United Kingdom, as well as capture-recapture and multiplier methods used in other countries.

## Supplementary Material

Supplementary Materials

## Figures and Tables

**Table 1:** Summary data by financial year and demographic group for fatal drug-related poisonings (fDRPs) and person-years at risk while on and off treatment in the Drug Data Warehouse (DDW), plus fDRPs not linked to the DDW (*d^miss^*) and total known opioid dependent people (*n^DDW^*). Data are aggregated across regions for display purposes only.

Year	Group	On treatment			Off treatment			Unobserved fDRPs (d^miss^)	Observed opioid- dependent people (n^DDW^)
		fDRPs (d^DDW^)	Person- years	Rate/ 1000	fDRPs (d^DW^)	Person- years	Rate/ 1000		
2005/06	Males < 35	55	32 454	1.69	42	12 160	3.45		
	Males 35–64	35	24 155	1.45	42	5945	7.07		
	Females < 35	14	15 506	0.90	5	4016	1.25		
	Females 35–64	9	8524	1.06	8	1643	4.87		
2006/07	Males < 35	50	36 073	1.39	33	14 389	2.29		
	Males 35–64	39	30 133	1.29	39	7988	4.88		
	Females < 35	10	17 223	0.58	7	4507	1.55		
	Females 35–64	9	10 484	0.86	8	2122	3.77		
2007/08	Males < 35	45	38 004	1.18	35	16 999	2.06		
	Males 35–64	72	36 063	2.00	32	10 042	3.19		
	Females < 35	11	18 113	0.61	5	4750	1.05		
	Females 35–64	16	12 262	1.30	5	2604	1.92		
2008/09	Males < 35	38	38 995	0.97	31	16 257	1.91	113	70 616
	Males 35–64	55	41 937	1.31	24	10 714	2.24	123	59 483
	Females < 35	9	18 815	0.48	0	4510	0.00	15	27 935
	Females 35–64	20	13 962	1.43	4	2742	1.46	22	18 295

NB: person-years do not sum to ‘Observed opioid-dependent people’ in 2008/09, as some individuals entered the risk set during the year.

## Box 1 Difficulties with capture–recapture estimation: case study of annual estimation in England

Capture-recapture analysis involves modelling the overlap between lists of individuals in contact with *K* services or bodies, usually using log-linear regression. The pattern of overlap is extrapolated to estimate the missing cell count—the number of individuals from the target population who were not observed in any of the data sources—and hence the prevalence. Although some level of dependencies between data sources can be accounted for by including interaction terms in the model, a key assumption is made that there is no K-way interaction. This has been described as an ‘act of faith’ and is impossible to verify from the data [38]. Caution about the validity of capture–recapture-based estimates is advised in particular if the ‘saturated model’ (a model that includes the maximum possible number of interaction terms) has the best fit after penalizing for model complexity [30]. When this is the case, alternative models with the same number of parameters and identical ‘fit’ can provide widely different prevalence estimates [23].

In practice, there are usually multiple potential sources of dependencies and heterogeneity in data sources used for capture–recapture. In particular, we have shown that cross-referral of individuals between drug treatment and other services—which may be a sign of a more developed approach to addressing opioid disorders—creates a complex dependence structure and potential bias [23]. For example, in England, official annual estimates of the number of opioid and problem drug users are based on capture–recapture analysis of the overlap between four data sources: ‘treatment’, ‘arrest for possession’, ‘probation’ and ‘prison’ [39,40]. The anticipated dependence structure between these is very complex, in particular due to direct referrals between the three criminal justice system sources and from any of these into treatment [23].

Given this complexity, it is not surprising that inclusion of multiple three-way interaction terms between data sources (e.g. the saturated model) is required to obtain adequate model fit, and that the possibility of a four-way interaction cannot be excluded. For example, we found this to be the case in a re-analysis of the problem drug use data set from 2009/10 [41]. Estimates from a number of models with the same fit were highly variable and unrealistically high, ranging from 2.1 to 6.6% of the population aged 15–64 years. See Supporting information, Appendix A for more detail. This is in stark contrast to the official estimate from the same year:0.89% (95% confidence interval = 0.87–0.92%) [41]. However, these annual estimates are dependent upon a number of unjustified decisions made in the model-fitting procedure. In particular, the analysts only allow a maximum of two (two-way) interaction terms, regardless of model fit statistics and despite the complex dependence structure described above. Three-way interaction terms are not considered. There is no theoretical basis for this rule, although it has been noted that researchers might ‘try to avoid fitting the saturated model’ because it often produces higher prevalence estimates [42]. Other authors have also demonstrated a tendency for capture–recapture models including higher order interactions to produce higher, and often extremely variable, prevalence estimates [43]. In the annual estimation exercise in England, subjective judgements are also made to replace any estimates not deemed to be ‘credible’ with estimates based on an alternative method [39]. We further note that analyses are stratified by local area and, in some areas, also by age group and gender, which reduces statistical power to detect dependencies between data sources. Prevalence estimates by local area, age group and gender are important for planning purposes, but a statistically more powerful approach is to generate these from a single regression model with covariates [44].
