# Supplementary material for: Estimating the prevalence of problem drug use from drug-related mortality data
Source: Addiction. Author manuscript; Available in PMC 2022 Dec 19. (PMC7613965; doi:10.1111/add.15111)
Supplement: Supplementary Materials [file EMS158434-supplement-Supplementary_Materials.docx]

**Supplementary materials**

**Appendix A: Re-analysis of 2009/10 problem drug use capture-recapture data**

Rather than fitting multiple stratified models, we fitted a single Poisson regression model to all of the data (1). In initial analyses, we aggregated the data across gender, age group and geographical areas, to obtain a contingency table of 2^4^– 1 = 15 counts (number of problem drug users observed in each combination of the 4 data sources). We used a simple model fitting procedure, starting with the simplest model and then introducing interactions between data sources only if the Akaike Information Criterion (AIC: a measure of model fit, penalising for complexity) was reduced. We found that the AIC was minimised by the saturated model, which includes all 2-way and all 3-way interactions between the 4 sources. This produced a prevalence estimate of 4.7% (95% confidence interval 3.4-6.6%). A selection of alternative saturated models with identical fit by definition (2) produced estimates ranging from 2.1 to 3.0%. Without additional information, it is impossible to choose between these conflicting estimates.

We then modelled the data stratified by age group (15-34 years, 35-64 years), gender (male, female) and region (nine Government Office Regions). We included covariates for these variables and interactions between these and each data source in the regression model. We also considered inclusion of 3-way interactions between covariates and data sources. This allows, for example, the strength of dependence between two data sources (e.g. treatment and prison) to vary across regions. We found that the AIC still supported inclusion of all 2-way and all 3-way interactions between data sources, and overall estimates remained unrealistically high (broadly similar to estimates from the aggregate analysis described above) from a range of models with similar fit.

**Appendix B: Details of the statistical model**

We estimate prevalence of opioid dependence in financial year (April to March) 2008/09 in each of 36 groups, defined by all possible combinations of gender, age group and region (as categorised in Appendix A).

We denote prevalence in group $g=1,...,36$by $\pi_{g}$. The absolute number of opioid-dependent people in each group is denoted by $N_{g}=P_{g}\pi_{g}$, where $P_{g}$is the total population size of group $g$, taken from official population size estimates.

In the Drug Data Warehouse (DDW) in 2008/09, we observe a number of opioid-dependent people, say $n_{g}^{DDW}$, in each group, $g$. We therefore only need to estimate the undercount of the DDW in each group, which we will denote by $n_{g}^{miss}=N_{g}-n_{g}^{DDW}$.

We define the following covariates for group:

$$age_{g}=\left\{\begin{matrix} -0.5\text{ } \text{if group }g\text{ 15-34 years} \\ 0.5\text{ if 35-64 years} \end{matrix}\right.$$

$$gender_{g}=\left\{\begin{matrix} -0.5\text{ if group }g\text{ male} \\ 0.5\text{ if female} \end{matrix}\right.$$

$$age\_gender_{g}=\left\{\begin{matrix} 0.5\text{ if group }g\text{ female and age 35-64 years} \\ -0.5\text{ otherwise} \end{matrix}\right.$$

We use $r(g)=1,...,9$to denote the region of group g.

1. **Model for fatal drug-related poisoning rate among Opioid-Dependent People**

To estimate prevalence from mortality data, we require estimates of fatal drug-related poisoning (fDRP) rates among opioid-dependent people in the same year (i.e. 2008/09). Although fDRP rates in years prior to this are not required for prevalence estimation, modelling multiple years of mortality data together in a single model increases precision in estimation.

We used data from the DDW to model fDRP rates among opioid-dependent people over a four-year period (financial years 2005/06 - 2008/09). We index year by $y=1,...,4$and also use an additional index $t=1,2$to denote observations during periods ‘off’ versus ‘on’ opioid substitution treatment respectively. We define the dummy variable: $treatment_{t}$= 0 (‘off treatment’, i.e. $t=1$), 1 (‘on treatment’, i.e. $t=2$).

Let $d_{gty}^{DDW}$denote the number of fDRPs observed during a period of $pyr_{gty}$person-years at risk, in group *g*, year *y* and with treatment status *t*. We assume

$$d_{gty}^{DDW}\sim Poisson(pmatch\times\lambda_{gty}pyr_{gty})$$

where $\lambda_{gty}$is the true fDRP rate among the relevant population and $pmatch$represents the probability that an fDRP in the DDW sample has been correctly identified, based on imperfect linkage to official mortality records (which was based only on initials and dates of birth). We assumed that this parameter was constant across groups, treatment status and year. Since fDRPs, particularly using our highly restrictive definition, are rare events, we assumed that all fDRPs linked to the opioid-dependent DDW sample were true positives.

Our final selected model for $\lambda_{gty}$was as follows:

[A1]

where $\beta_{1},\beta_{2},\beta_{3},\beta_{4},\beta_{5}$represent (respectively): main effect of age, main effect of gender, age by gender interaction, main effect of treatment status, and age group by treatment status interaction. The parameters $\delta_{y}$ and represent main effects of year, and year by treatment interactions. Coefficients for the baseline year, $\delta_{1}$and , were set to zero. Regional effects are represented by $\zeta_{r}$, r = 1,…,9.

It was not feasible to estimate the region effects $\zeta_{r}$independently in each region, due to the sparsity of the mortality data. We therefore fitted $\zeta_{r}$ as random effects, assumed to be normally distributed across regions, with a mean of zero: $\zeta_{r}\sim Normal(0,\tau_{\zeta}^{2}),\text{ }r=1,...,9$.

Vague $Normal(0,100)$ prior distributions were assumed for $\beta_{1},...,\beta_{5}$,$\delta_{2},\delta_{3},\delta_{4}$ and .. The intercept term,$\beta_{0}$, was assigned a $Normal(-6,100)$prior distribution, corresponding to centring around an fDRP rate among opioid-dependent people out of treatment in 2005/06 of 2.5 fDRPs per 1000 person years (roughly the observed untreated mortality rate), but with a very large variance. We assigned a vague $Uniform(0,5)$ prior distribution to the between-region standard deviation parameter, $\tau_{\zeta}$.

It is not possible to estimate $pmatch$from the data, so it was necessary to set an informative prior distribution for this parameter. We assumed$pmatch\sim Uniform(0.74,0.84)$, where 74% and 84% are lower and upper plausible bounds that we derived based on coding of mortality in treatment records among the DDW sample.

In a sensitivity analysis, we explored the effect of removing the interaction between treatment and year (): see manuscript, Section 4.2.

1. **Model for fDRPs not linked to the DDW data**

Let $d_{g}^{miss}$denote the number of fDRPs among group $g=1,...,16$that were *not* linked to the DDW data. We use these data to estimate $n_{g}^{miss}$and therefore prevalence, $\pi_{g}$.

By definition, these $n_{g}^{miss}$individuals were all ‘out’ of treatment throughout the entirety of 2008/09, otherwise they would have been observed in the DDW data. They therefore contribute $n_{g}^{miss}$person-years at risk. We assume that fDRP rates in these individuals are equal to $\lambda_{g14}$, i.e. the mortality rate among opioid dependent people in group *g*, during periods *out* of treatment, in the relevant year (year 4: 2008/09), estimated from the mortality rate model above.

We assume that:

$$d_{g}^{miss}\sim Poisson\left(\lambda_{g14}n_{g}^{miss}+(1-pmatch)(\lambda_{g14}pyr_{g14}+\lambda_{g24}pyr_{g24})\right)$$

If matching of DDW data to mortality records were perfect ($pmatch=1$) then the expectation of $d_{g}^{miss}$would be $\lambda_{g14}n_{g}^{miss}$. However, as matching is imperfect, we add two additional terms to the expectation of $d_{g}^{miss}$, representing deaths in the DDW sample during the observed person-years at risk, which were missed due to incomplete matching: $(1-pmatch)(\lambda_{g14}pyr_{g14}+\lambda_{g24}pyr_{g24})$

1. **Model for the prevalence of opioid dependence**

We assumed the following linear model for logit-transformed prevalence in each group, $g$:

$$\text{logit}(\pi_{g})=\alpha_{r(g)}+\gamma_{1}age_{g}+\gamma_{2}gender_{g}+(\gamma_{3}age\_gender_{g})+\upsilon_{r(g)}age_{g}+\omega_{r(g)}gender_{g}$$

The region-specific intercept terms$\alpha_{1},...\alpha_{9}$were estimated independently: each was assigned a $Normal(-4.6,100)$prior distribution. This corresponds to centring around an overall prevalence in each region of 1%, but with a very wide variance. As a sensitivity analysis, we re-ran the model with a prior mean of -3.2, corresponding to an unrealistically high prevalence in each region of 4%. Results were robust to this change: e.g. the overall prevalence estimate (%) with 95% Cr-I were the same to 2 decimal places

The parameters $\upsilon_{r(g)}$ and $\omega_{r(g)}$represent interactions terms between region and age group, and between region and gender respectively. Due to sparsity of data, these could not be estimated independently across regions. We therefore modelled these as random effects, assumed normally distributed across regions with a mean of zero: $\upsilon_{r}\sim Normal(0,\tau_{\upsilon}^{2}),\text{ }\omega_{r}\sim Normal(0,\tau_{\omega}^{2}),\text{ }r=1,...,9$.

We assigned vague $Normal(0,100)$ prior distributions to the fixed effect regression coefficients $\gamma_{1},\gamma_{2},\gamma_{3}$and $Uniform(0,5)$ prior distributions to the two between-region standard deviation parameters, $\tau_{\upsilon}$and $\tau_{\omega}$.

We also fitted an extended version of this model including three-way interactions between gender, age group and region (also modelled as random effects). This model resulted in an increase in DIC of 0.8 and no appreciable change in estimates, so these interaction terms were not retained.

1. **Model fitting**

Models were fitted using the Bayesian software, WinBUGS, which estimates posterior distributions of parameters through Markov chain Monte Carlo simulation.

We enforced a constraint in the model to ensure that $n_{g}^{miss}$ is non-negative for all $g$. This was achieved by inputting into the model code the assumption of a distribution to each $n_{g}^{DDW}$. The probability parameters $p_{g}^{DDW}$are ‘nuisance parameters’ (not of interest in their own right). These were each assigned uninformative $Uniform(0,1)$prior distributions.

Presented results are based on running two parallel chains, for 5 million iterations, after having discarded a burn-in period of 500,000 iterations. Chains were “thinned” to include every 50^th^ iteration when storing and summarising posterior distributions.

Convergence and mixing were assessed using the Gelman-Rubin diagnostic and visual inspection of chain histories. The chains exhibited substantial autocorrelation, in particular for the prevalence model parameters, hence the long model runs to ensure stability of results.

The overall model fit appeared to be good, with a posterior mean residual deviance of 305.1 on 324 unconstrained data points (Table A1). A well fitting model can be expected to have posterior mean residual deviance close to the number of data points (3). The removal of the *year X treatment* interaction term in the mortality model (sensitivity analysis described in Section 4.2) led to an increase in DIC by 9.8 points. Omission of other interaction terms also resulted in a substantially worse fit, while addition of other interaction terms (for instance, *year* X *group* or *year* X *region* in the mortality model) provided negligible improvement in model fit.

Table A1. Model fit statistics for each level of model aggregation. N = number of observations at the given aggregation level; pD = effective number of parameters; Deviance = posterior mean deviance.

|  |  | Data type: | | | Total |
| --- | --- | --- | --- | --- | --- |
| Aggregation level | Statistic | fDRPs in DDW cohort in treatment $(d^{DDW})$ | fDRPs in DDW cohort out of treatment $(d^{DDW})$ | fDRPs not linked to the DDW cohort* $(d^{miss})$ |  |
| Full  (*year* *X* *region* *X* *gender X age*) | N | 144 | 144 | 36 | 324 |
|  | pD | 10.2 | 8.1 | 17.7 | 36.0 |
|  | Deviance | 137.6 | 138.0 | 29.5 | 305.1 |
| *year X region* | N | 36 | 36 | 9 | 81 |
|  | pD | 8.0 | 6.6 | 8.1 | 22.7 |
|  | Deviance | 30.5 | 37.1 | 8.5 | 76.1 |
| *region* *X* *gender X age* | N | 36 | 36 | 36 | 108 |
|  | pD | 7.2 | 5.2 | 17.7 | 30.1 |
|  | Deviance | 35.8 | 32.6 | 29.5 | 97.9 |
| *year* *X gender X age* | N | 16 | 16 | 4 | 36 |
|  | pD | 6.2 | 5.5 | 3.3 | 15.0 |
|  | Deviance | 18.7 | 17.6 | 3.3 | 39.6 |
| *For year 4 only - *region* X *gender X age* | | | | | |

**Appendix C: WinBUGS model code**

Data are in "long" format, index by year[] = 1,…,4, reg[] = 1,…,9 (i.e. region) and group[] = 1,…,4.

Total number of rows of data = 4*9*4 = 144.

model{

#######################

**# MORTALITY MODEL #**

#######################

for(i in 1:144){

## Likelihood:

d_ddw_outtrt[i] ~ dpois(pred_d_ddw_outtrt[i]) # Out of treatment fDRP

d_ddw_intrt[i] ~ dpois(pred_d_ddw_intrt[i]) # In treatment fDRP

pred_d_ddw_outtrt[i] <- pmatch * lambda_outtrt[i] * pyr_outtrt[i]

pred_d_ddw_intrt[i] <- pmatch * lambda_intrt[i] * pyr_intrt[i]

## Regression model for out of treatment fDRP rate:

log(lambda_outtrt[i]) <- beta0 + beta[1]*age.g[group[i]] + beta[2]*sex.g[group[i]] + beta[3]*agesex.g[group[i]]

+ delta[year[i]] + zeta[reg[i]]

## Regression model for in treatment fDRP rate log(lambda_intrt[i]) <- log(lambda_outtrt[i])

+ beta[4] + beta[5]*age.g[group[i]] + phi[year[i]]

}

## Priors for mortality model:

## intercept

beta0 ~ dnorm(-4.8, 0.01)

## Main effects age, gender, treatment and interactions between age x gender, age x treatment

for(j in 1:5){

beta[j] ~ dnorm(0, 0.01)

}

## year effect and treatment x year interaction

delta[1] <- 0

phi[1] <- 0

for(y in 2:4){

delta[y] ~ dnorm(0, 0.01)

phi[y] ~ dnorm(0, 0.01)

}

## Regional random effect

for(r in 1:9){

zeta[r] ~ dnorm(0, prec.zeta)

}

sd.zeta ~ dunif(0, 5)

prec.zeta <- pow(sd.zeta, -2)

# Proportion of true DDW deaths that were correctly linked to ONS deaths register:

pmatch ~ dunif(0.74, 0.88)

#####################

# **PREVALENCE MODEL #**

#####################

# Only model year 4: last 36 rows of data

for(i in 109:144){

## Likelihood:

d_miss[i] ~ dpois(pred_d_miss[i])

pred_d_miss[i] <- lambda_outtrt[i] * nmiss[i]

+ (1 - pmatch) * (lambda_outtrt[i] * pyr_outtrt[i] + lambda_intrt[i] * pyr_intrt[i])

nmiss[i] <- N[i] - n_ddw[i]

N[i] <- P[i] * pi[i]

## Model for total prevalence - fixed region intercepts

logit(pi[i]) <- gamma[1]*age.g[group[i]] + gamma[2]*sex.g[group[i]]

+ gamma[3]*agesex.g[group[i]]

+ alpha[reg[i]] + upsilon[reg[i]] * age.g[group[i]]

+ omega[reg[i]] * sex.g[group[i]]

## Ensuring that the number of known problem drug users is positive

n_ddw[i] ~ dbin(p_ddw[i],N[i])

p_ddw[i] ~ dunif(0,1)

}

## Priors for prevalence model:

## main effects of age and sex and age x sex interaction

for(j in 1:3){

gamma[j] ~ dnorm(0, 0.01)

}

for(r in 1:9){

## fixed region intercepts - replaces overall intercept

alpha[r] ~ dnorm(-4.6, 0.01)

## regional random effects for age

upsilon[r] ~ dnorm(0, prec.upsilon)

## regional random effects for sex

omega[r] ~ dnorm(0, prec.omega)

}

sd.omega ~ dunif(0, 5)

sd.upsilon ~ dunif(0, 5)

prec.omega <- pow(sd.omega, -2)

prec.upsilon <- pow(sd.upsilon, -2)

#####################################

# Estimated total opioid-dependent people #

# by different levels of aggregation #

#####################################

## Total N by region, gender/age group and overall:

N.group[1] <- N[109]+ N[113]+ N[117]+ N[121]+ N[125]+ N[129]+ N[133]+ N[137]+ N[141]

N.group[2] <- N[110]+ N[114]+ N[118]+ N[122]+ N[126]+ N[130]+ N[134]+ N[138]+ N[142]

N.group[3] <- N[111]+ N[115]+ N[119]+ N[123]+ N[127]+ N[131]+ N[135]+ N[139]+ N[143]

N.group[4] <- N[112]+ N[116]+ N[120]+ N[124]+ N[128]+ N[132]+ N[136]+ N[140]+ N[144]

N.reg[1] <- sum(N[109:112])

N.reg[2] <- sum(N[113:116])

N.reg[3] <- sum(N[117:120])

N.reg[4] <- sum(N[121:124])

N.reg[5] <- sum(N[125:128])

N.reg[6] <- sum(N[129:132])

N.reg[7] <- sum(N[133:136])

N.reg[8] <- sum(N[137:140])

N.reg[9] <- sum(N[141:144])

# Total N and total missing:

Ntot <- sum(N[109:144])

nmisstot <- sum(nmiss[109:144])

}

1. Tilling K, Sterne JAC. Capture-recapture models including covariate effects. American Journal of Epidemiology. 1999;149(4):392-400.

2. Jones HE, Hickman M, Welton NJ, De Angelis D, Harris RJ, Ades AE. Recapture or precapture? Fallibility of standard capture-recapture methods in the presence of referrals between sources. Am J Epidemiol. 2014;179(11):1383-93.

3. Welton NJ. Evidence synthesis for decision making in healthcare. Chichester, West Sussex: John Wiley & Sons; 2012. xii, 282 p. p.
